# The Optimal Fluid Strategy Matters in Liver Surgery: A Retrospective Single Centre Analysis of 666 Consecutive Liver Resections

**DOI:** 10.3390/jcm12123962

**Published:** 2023-06-10

**Authors:** Katharina Hoeter, Stefan Heinrich, Daniel Wollschläger, Felix Melchior, Anna Noack, Verena Tripke, Hauke Lang, Serge C. Thal, Dorothee H. Bremerich

**Affiliations:** 1 Department of Anaesthesiology, University Medical Centre of the Johannes Gutenberg-University, 55131 Mainz, Germany; 2 Department of General, Visceral and Transplantation Surgery, University Medical Centre of the Johannes Gutenberg-University, 55131 Mainz, Germany; stefan.heinrich@unimedizin-mainz.de (S.H.);; 3 Institute of Medical Biostatistics, Epidemiology and Informatics (IMBEI), University Medical Centre of the Johannes Gutenberg-University, 55131 Mainz, Germany

**Keywords:** intravenous infusion, fluid management, surgical procedures, hepatectomy, adverse effects

## Abstract

As optimal intraoperative fluid management in liver surgery has not been established, we retrospectively analyzed our fluid strategy in a high-volume liver surgery center in 666 liver resections. Intraoperative fluid management was divided into very restrictive (<10 m kg^−1^ h^−1^) and normal (≥10 mL kg^−1^ h^−1^) groups for study group characterization. The primary endpoint was morbidity as assessed by the Clavien–Dindo (CD) score and the comprehensive complication index (CCI). Logistic regression models identified factors most predictive of postoperative morbidity. No association was found between postoperative morbidity and fluid management in the overall study population (*p* = 0.89). However, the normal fluid management group had shorter postoperative hospital stays (*p* = <0.001), shorter ICU stays (*p* = 0.035), and lower in-hospital mortality (*p* = 0.02). Elevated lactate levels (*p* < 0.001), duration (*p* < 0.001), and extent of surgery (*p* < 0.001) were the most predictive factors for postoperative morbidity. In the subgroup of major/extreme liver resection, very low total (*p* = 0.028) and normalized fluid balance (*p* = 0.025) (NFB) were associated with morbidity. Moreover, fluid management was not associated with morbidity in patients with normal lactate levels (<2.5 mmol/L). In conclusion, fluid management in liver surgery is multifaceted and must be applied judiciously as a therapeutic measure. While a restrictive strategy appears attractive, hypovolemia should be avoided.

## 1. Introduction

Liver surgery has become a safe treatment for primary and secondary liver tumors due to refinements in parenchymal transection and improved perioperative management. Compared to intestinal surgery, liver surgery is generally associated with higher blood loss, different metabolic stress responses, and organ-specific complications. In addition, liver surgery has higher morbidity and mortality rates compared to other surgical patient populations (e.g., colorectal surgery) [1,2]. Inflow occlusion (Pringle maneuver) is an established measure to prevent bleeding during parenchymal transection. As liver ischemia may cause significant liver injury and impaired liver function, most surgeons prefer intermittent inflow occlusion during prolonged transection phases [3]. Total vascular exclusion (TVE) in addition to inflow occlusion or ante-situm resections completely prevents bleeding and allows reconstruction of hepatic veins [4]. However, these procedures affect the patient’s circulatory status and increase the requirements for optimal anesthetic management during liver surgery. Consequently, these useful procedures should only be used when necessary.

More than 20 years ago, a low central venous pressure (CVP) strategy was identified as a strategy to reduce blood loss during liver surgery [5], since a lower CVP is considered to be associated with lower pressures in the venous system of the liver, resulting in less blood loss, particularly during inflow occlusion periods. Since then, this low-CVP strategy has been adopted by many expert centers, and a recent survey among expert centers for liver surgery revealed that more than 60% of respondents follow this low-CVP strategy. However, fluid management varies widely among these centers, with 22% following goal-directed therapy and 6% aiming for euvolemia to minimize blood loss [6].

The enhanced recovery after surgery (ERAS) society also recommends balanced fluid management with the goal of euvolemia in liver surgery [1]. According to the ERAS recommendations, balanced crystalloids should be preferred over saline as the primary fluid replacement, and a low-CVP strategy is also recommended to reduce blood loss [1].

However, optimal perioperative fluid management in patients undergoing major abdominal and, in particular, liver surgery is still under debate. Parameters to guide intraoperative fluid management have not yet been defined. Excessive fluid administration may compromise oxygenation [7] due to hemodilution, cause pulmonary complications [8], and increase the risk of wound infection and anastomotic leakage due to intestinal edema and impaired collagen regeneration. On the other hand, intraoperative hypovolemia can impair cardiac output and lead to hypoperfusion and organ dysfunction [9]. Current research mainly focuses on comparing liberal, restrictive, and goal-directed fluid management, with conflicting results [10,11,12,13].

The aim of this study was to define solid parameters for optimal fluid management during general anesthesia for patients undergoing liver resection. Therefore, we compare the outcomes of patients with regard to intraoperative fluid management at a high-volume liver surgery center where general anesthesia is based on a low-fluid/low-CVP strategy.

## 2. Materials and Methods

### 2.1. Procedures

The data from patients who underwent liver resection at the Department of General, Visceral, and Transplantation Surgery of the University Medical Centre Mainz between December 2014 and September 2018 were analyzed. The primary data source was the hospital’s digital clinical database. The electronic health records were searched for operating codes 5-501 and 5-502 for liver procedures. Data were collected primarily to evaluate the influence of intraoperative fluid management on postoperative outcome in patients undergoing liver resection. Other risk factors for postoperative adverse events related to anaesthesiological management were also considered.

The ethics committee of the Medical Association of the State of Rhineland–Palatine (Germany) approved this retrospective study (registration number: 2020-14894-retrospective) and waived the requirement for informed consent.

### 2.2. Eligibility Criteria

**Figure 1:**
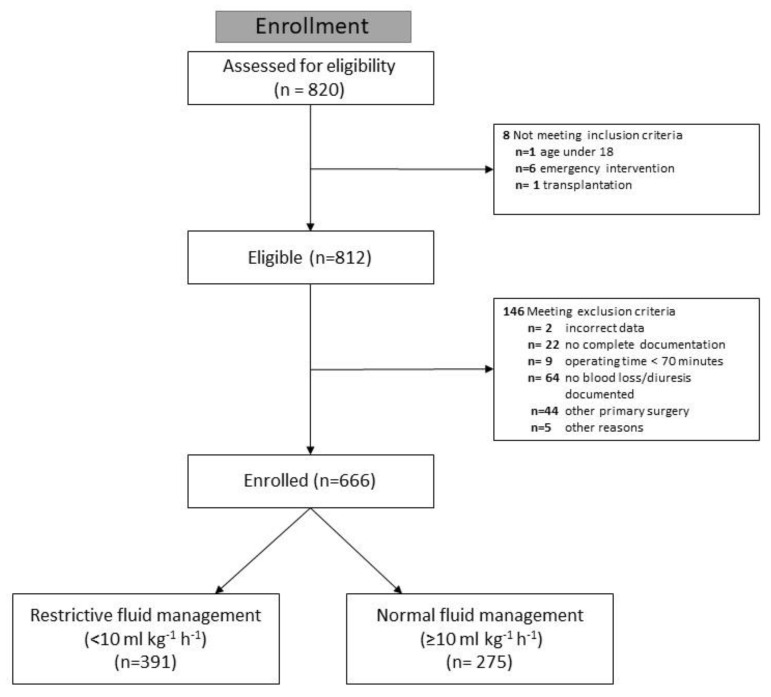
CONSORT Flow chart of patient eligibility.

Patients aged at least 18 years who underwent liver resection were eligible for analysis. We excluded liver transplantations and emergency procedures, as well as resections as part of other primary surgical procedures, due to special hemodynamic preconditions. We also excluded liver resections with an operative time of less than 70 min, incomplete documentation of intraoperative fluid administration, estimated blood loss, or diuresis (Figure 1). Only the first procedure for each patient in the study was analyzed, and no subsequent procedures during hospitalization were included.

### 2.3. Study Design

This is a retrospective analysis. The anesthesia team continuously documented intraoperative anesthesiological parameters (heart rate, blood pressure, fluid and drug administration, and diuresis).

### 2.4. Perioperative Management

Patients scheduled for liver resection do not receive specific preoperative measures at our center. General anesthesia is routinely induced with propofol- and sufentanil-based injection anesthesia and maintained with balanced sevoflurane inhalation anesthesia. In general, fluid restriction with euvolemia and a low-CVP strategy are anticipated, and a decrease in arterial blood pressure of >20% is avoided. There was no standard operating procedure for fluid management in patients undergoing liver resection. Therefore, the intraoperative management decisions are made by the attending anesthesiologists, with potential patient-to-patient variability in the anesthesiological management based on provider preference. In general, balanced acetate and malate-buffered Sterofundin^®^ ISO (B. Braun, Melsungen, Germany) is used as the standard crystalloid fluid according to ERAS recommendations [1]. For colloid fluid replacement, Hemohes^®^ 6% (B. Braun, Melsungen, Germany), Voluven^®^ 6% (Fresenius Kabi, Germany), or gelatine solutions such as Gelafundin^®^ ISO 40 mg mL^−1^ infusion solution (B. Braun, Melsungen, Germany) are administered. Intraoperative monitoring parameters, such as heart rate registration, invasive and non-invasive blood pressure measurements, SpO2 (% hemoglobin saturation), 5-channel echocardiogram, CVP, diuresis, estimated blood loss, and temperature (°Celsius), are used according to clinical standards. A pringle maneuver is applied on demand using an intermittent clamping strategy. Tissue dissection is usually performed mechanically using scissors or a crush-clamp technique. Hilar structures and hepatic veins are transected using vascular clamps and closed with prolene sutures. Postoperatively, patients are routinely monitored overnight in the recovery room. Depending on comorbidities, the extent of surgery, and the intraoperative course, patients may be transferred to the intermediate care unit (IMC) or intensive care unit (ICU). Patients are transferred to the regular ward the following day if there is no evidence of bleeding and hemodynamic support is not required. Pain management is based on intravenous analgesics such as metamizole and piritramid. Epidural anesthesia is not used for liver surgery.

### 2.5. Extent of Surgery

Liver resections involving ≤3 segments were considered minor, whereas those involving four or more segments were considered major. Major resections with additional procedures such as portal vein or bile duct resection/reconstruction were categorized as extreme liver surgery.

### 2.6. Intraoperative Fluid Management

Total intraoperative total fluid balance (TFB) was calculated using the following formula:

(Crystalloid-infusion + colloid-infusion) − (estimated blood loss + diuresis).

The total fluid balance was normalized for anesthesia time and patient weight (NFB).

$$\frac{\left(Crystalloid-infusion+colloid-infusion)-(estimated\;blood\;loss+diuresis\right)}{kilogramm\;bodyweight\times ansthetic\;time\;(hours)}$$

Based on this intraoperative fluid management, patients were assigned to either a very restrictive fluid management group (<10 mL kg^−1^ h^−1^) or a normal fluid management group (≥10 mL kg^−1^ h^−1^) for study group characterization.

The number of intraoperatively administered red blood cell units (220–330 mL unit^−1^; hematocrit: 50–70%, with a tolerated deviation of 5%) was also documented. However, administration of erythrocytes, fresh frozen plasma (FFP), and platelets was not considered, as the volume is not standardized and can vary by 25–30%.

### 2.7. Hemodynamics

Intraoperative blood pressure was analyzed at 5-min intervals, and hypotension was defined as a decrease in systolic arterial blood pressure below 80% of baseline. Total hypotension time was calculated for each patient based on the number and duration of hypotensive episodes. The intraoperative pharmacological vasoactive support, e.g., norepinephrine, the highest intraoperative lactate level, and the last intraoperative hemoglobin level were also recorded.

In a further step, a more sensitive hypotension limit of mean arterial pressure (MAP) ≤ 65 mmHg was established, and the influence on postoperative morbidity was investigated according to the same protocol.

### 2.8. Surgical Morbidity

Postoperative morbidity based on the Clavien–Dindo (CD) classification is prospectively graded and documented in the surgical unit [14]. In addition, surgical morbidity was retrospectively re-evaluated from patient records to achieve a complete assessment. Briefly, the CD-classification grades each complication according to the extent of treatment required for the treatment of that complication: grade III complications require intervention, grade IV complications require intensive care treatment, and grade V defines the death of the patient. The CD-classification was applied for postoperative respiratory, hepatic, gastrointestinal, urologic, cardiac, circulatory, and neurologic adverse events, as well as infections and delirium.

For the analysis, the highest complication was considered for each patient, and the comprehensive complication index (CCI) was calculated, as a patient may have had several complications [15].

### 2.9. Statistical Analysis

The data were collected and independently checked for data entry errors by four authors. Data were analyzed using the statistical software R (R Core Team 2022: A language and environment for statistical computing, R Foundation for Statistical Computing, Vienna, Austria), version 4.2.2., and presented as mean ± SD or median for continuous variables and as frequencies and percentages for categorical variables. The primary endpoint was postoperative morbidity during hospitalization as assessed by the CD-classification (0 vs. 1–5) in any organ system. Secondary outcomes included morbidity assessed by the CCI, postoperative hospital and ICU length of stay, and in-hospital mortality. The postoperative hospital length of stay was measured from the date of surgery to hospital discharge. For the purpose of simple group comparison, patients with intraoperative NFB < 10 mL kg^−1^ h^−1^ were categorized as having received very restrictive fluid management, whereas those with ≥10 mL kg^−1^ h^−1^ were categorized as having received normal fluid management. Logistic regression was used to predict a CD sum score of >0 vs. =0 from intraoperative fluid balance as a continuous risk factor, adjusting for sex, age, extent as well as duration of surgery, crystalloid infusion, last hemoglobin-level (<10 vs. ≥10 g/dL), highest intraoperative lactate level (<1.5 vs. ≥1.5 mmol∙L^−1^), norepinephrine, red blood cell transfusion, estimated blood loss, time of relative hypotension (<100 vs. ≥100 min), and ASA physical status (I/II vs. III/IV). Linear regression modelling was used to examine intraoperative fluid management as a continuous risk factor for postoperative complications graded by the CCI, adjusting for the same set of covariates. The association of postoperative length of stay with intraoperative fluid management was analyzed using Cox regression, adjusting for the same set of covariates. For patients admitted to the ICU, the association between ICU length of stay and intraoperative fluid management was examined using Cox regression with the same set of covariates. For these analyses, all IMC and ICU days per patient were summed, whereas overnight observation in the recovery room was not considered an ICU stay. Logistic regression for in-hospital mortality used continuous intraoperative fluid balance as a risk factor, adjusting for age, extent, duration of surgery, and crystalloid infusion rates. Here, the number of possible covariates was limited by the number of observed deaths. Linear regression for the log of norepinephrine normalized to patient weight and duration of anesthesia was used to examine the association of pharmacological vasoactive support with intraoperative fluid management, controlling for age. Hypotheses regarding intraoperative fluid management as a risk factor for postoperative complications were predefined. Additional exploratory analyses were performed to examine other risk factors for postoperative surgical adverse events. Finally, subgroup analyses were performed using logistic regression models for major and extreme liver resections and for the subgroup of normal lactate levels at the end of surgery for the same endpoints.

## 3. Results

We identified 820 patients scheduled for liver resection during the study period, of whom 666 met the eligibility criteria for this analysis (Figure 1).

Of these, 391 patients were assigned to the very restrictive (<10 mL kg^−1^ h^−1^) and 275 patients to the normal (≥10 mL kg^−1^ h^−1^) groups. Patient demographics and characteristics are summarized in Table 1. The standardized mean difference of NFB between ASA groups I/II vs. III/IV is presented as Cohen’s d (0.25, 95% confidence interval 0.09–0.40).

Most resections involved less than four segments (minor), and one third of the resections were major/extreme resections (35.5%).

**Figure 2:**
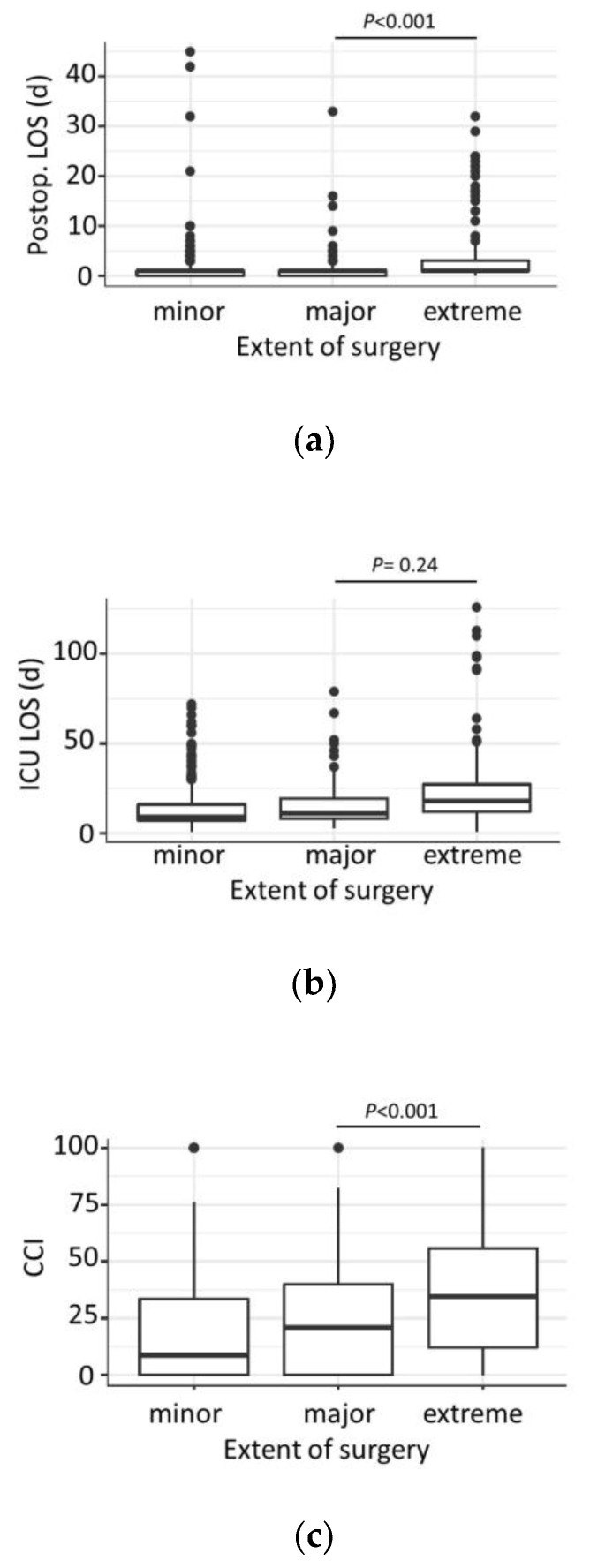
Subgroup analysis on extent of surgery: (**a**) Hospital length of stay (LOS) and extent of surgery; (**b**) ICU length of stay (LOS) and extent of surgery; (**c**) Comprehensive complication index (CCI) and extent of surgery.

The duration of surgery, postoperative hospital length of stay (Figure 2a), and ICU length of stay (Figure 2b) gradually increased with the extent of liver surgery.

### 3.1. Intraoperative Circulatory Parameters and Fluid Management

In general, older patients required higher doses of norepinephrine (*p* = 0.001) than younger patients. In addition, higher blood loss was associated with higher norepinephrine doses (*p* < 0.001).

The mean intraoperative NFB was 9.77 ± 5.24 mL kg^−1^ h^−1^ and ranged from −2.71 to 57.4 mL kg^−1^ h^−1^. NFB gradually decreased with the extent of surgery and was associated with blood loss (*p* < 0.001). Intraoperative NFB showed only an insignificant (*p* = 0.86) association with pharmacological vasoactive support.

**Figure 3:**
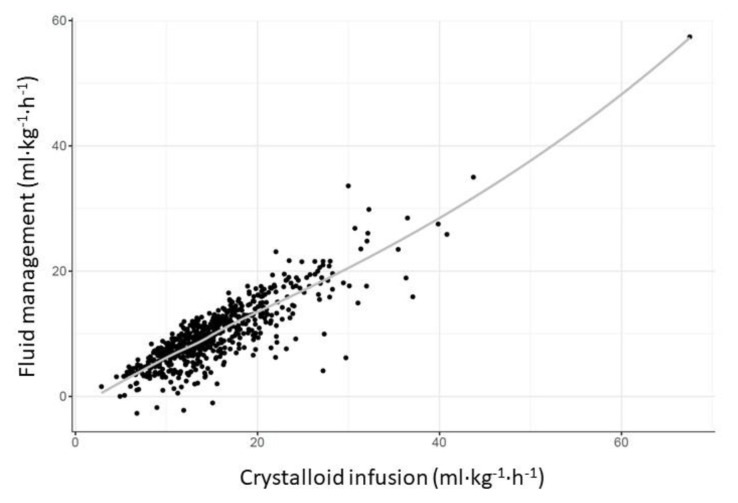
Association between NFB and crystalloid infusion. NFB was mainly based on crystalloid infusion, with a positive correlation between the two values.

A higher NFB was mainly due to a higher crystalloid infusion (19.5 ± 6.22 mL kg^−1^ h^−1^) compared to patients in the very restrictive group (11.9 ± 3.56 mL kg^−1^ h^−1^) (Table 1, Figure 3).

Patients in the normal group received 4500 mL (1500–24,000 mL) of crystalloids, in contrast to 4000 mL (1100–14,500 mL) in the very restrictive group. The total crystalloid infusion rate gradually increased with the extent of surgery, with minor liver resections receiving 2723 mL (−600–8700 mL), major resections receiving 3203 mL (100–8100 mL), and extreme resections receiving the highest with 3884 mL (−500–13,000 mL, *p* = 0.09).

### 3.2. Hospital Stay

The median postoperative hospital length of stay for the entire cohort of patients was 11 days (1–126 days), and 393/666 (59.9%) of patients required an ICU stay during the post-operative period for a median of 1 day (range 1–45 days).

The extent (*p* < 0.001) and duration of liver surgery (*p* < 0.001) were associated with a longer hospital length of stay. While higher total (*p* = 0.004) and NFB (*p* < 0.001, Table 2) were associated with a shorter hospital length of stay, higher volumes of crystalloid infusions (*p* = 0.004) were associated with a longer hospital length of stay (Table 3).

### 3.3. ICU Stay

Higher intraoperative lactate levels (*p* = 0.008), a shorter duration of surgery (*p* < 0.002), and a lower NFB (*p* = 0.035, Table 2) were associated with a longer ICU length of stay (Table 3).

### 3.4. Postoperative Morbidity

Regarding post-operative morbidity, approximately half of the patients (52.25%) had a post-operative complication, the majority of which were grade III (21.5%). The median CCI was 20.9.

The extent (*p* < 0.001) and duration of surgery (*p* < 0.001) were associated with morbidity: the median CCI gradually increased with the extent of surgery, as minor liver resections had a mean CCI of 18.5 (0–100), whereas major and extreme resections had median CCIs of 23.6 (0–100) and 40.3 (0–100), respectively (Figure 2C).

**Figure 4:**
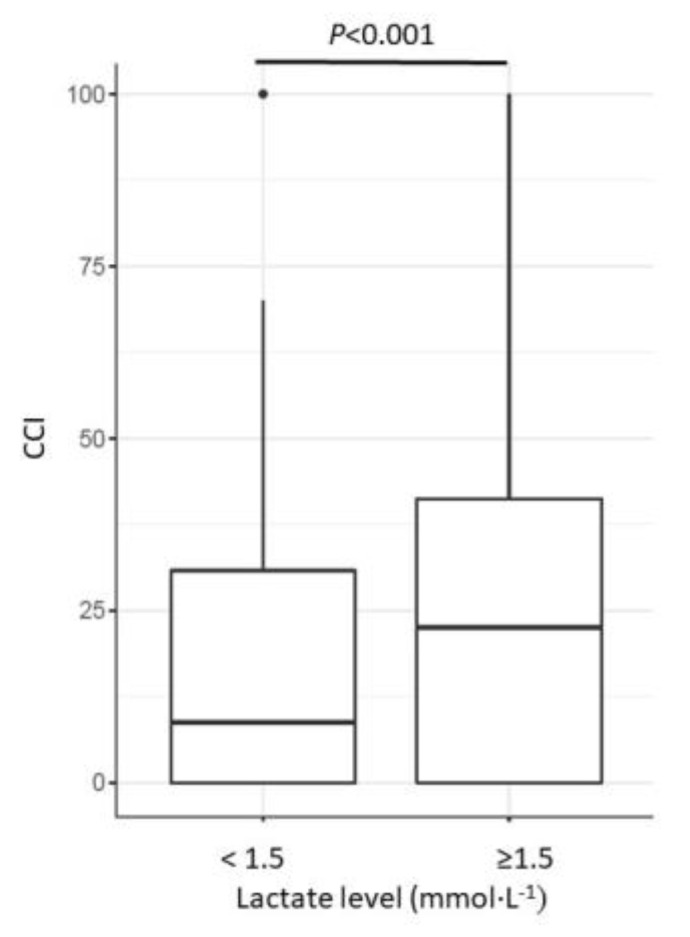
Association of intraoperative lactate levels and surgical morbidity (CCI). Patients with higher lactate levels revealed higher morbidity, according to the CCI (*p* < 0.001).

Increased intraoperative lactate levels (*p* < 0.001, Figure 4), red blood cell transfusion (*p* < 0.001), estimated blood loss (*p* < 0.001), and intraoperative vasoactive support with norepinephrine (*p* = 0.02) were associated with higher morbidity. We found no association of morbidity with the last intraoperative hemoglobin level (*p* = 0.022), the time of relative hypotension (*p* = 0.29), or intraoperative NFB (*p* = 0.89) (Table 4).

### 3.5. Mortality

Overall, 30 of the 666 (4.5%) patients died. As expected, major/extreme liver resections were associated with a higher mortality rate (*p* < 0.001). Interestingly, a very low NFB was also associated with a higher mortality rate (*p* = 0.023) compared to the total population in the study (*p* = 0.02, Table 2).

### 3.6. Hypotension

Additionally, after applying a MAP ≤65 mmHg as the cut-off for hypotension in the regression analysis, we did not find a significant association with postoperative complications as measured by the CD score (*p* = 0.10).

In this analysis, we found a significant association between NFB and CD score (*p* = 0.026). Again, NFB and CCI were not significantly associated (*p* = 0.23).

Moreover, NFB was again significantly associated with perioperative mortality (*p* = 0.023) and length of hospital stay (*p* = 0.0057). However, the length of the ICU stay was no longer significantly associated with NFB.

### 3.7. Subgroup Analyses

#### 3.7.1. Major/Extreme Liver Resections

As we expected the fluid strategy to be most relevant to the outcome of major/extreme liver resections, we performed subgroup analyses by excluding minor liver resections.

The median CCI of the major/extreme resections (n = 236) was 33.5 (0–100), with a mortality rate of 9.3%.

In major/extreme liver resections, patient age (*p* = 0.002), duration of surgery (*p* < 0.002), as well as very low total (*p* = 0.028), NFB (*p* = 0.025), and crystalloid fluid supplementation (*p* = <0.0001), were associated with a higher CCI. In this subgroup of patients, the highest lactate level (*p* = 0.007), the dose of norepinephrine (*p* = 0.004), the amount of blood loss (*p* = 0.003), and red blood cell transfusions (*p* < 0.001) were also associated with the CCI.

For the other parameters, the duration of surgery (*p* = 0.01) and the use of crystalloids (*p* = 0.02) were associated with a longer hospital length of stay.

In contrast to the overall cohort, only the duration of surgery (*p* < 0.0001) was an independent factor associated with a longer ICU length of stay in this subgroup analysis.

Regarding mortality, only the use of crystalloids was significantly associated with mortality (*p* = 0.01).

#### 3.7.2. Normal Serum Lactate Levels

We found an association between a very low TFB and a longer postoperative hospital length of stay, a longer ICU LOS, and a higher postoperative mortality rate. To analyze whether our general fluid management may have been too restrictive, and hypovolemia is often associated with elevated lactate levels, we excluded patients with elevated lactate levels and focused on patients with normal serum lactate levels (<2.5 mmol L^−1^).

In this subgroup of 471 patients, the mortality rate was 2.1% (10/471).

Neither morbidity nor ICU length of stay were associated with NFB or crystalloid balance in this subgroup. We only found a longer hospital length of stay following liberal fluid management (*p* = 0.01) in this subgroup. In addition, duration of surgery remained a significant factor for morbidity (*p* < 0.001), length of hospital stay (*p* < 0.001), and ICU (*p* < 0.001) stay.

Due to the very low mortality rate, we could not perform regression analyses for potential risk factors for mortality.

## 4. Discussion

Intraoperative fluid management has changed dramatically over the past 20 years, with increasing evidence that fluid overload increases surgical morbidity and mortality. This effect has been demonstrated for several surgical procedures, most notably colorectal surgery, and many studies suggest that restrictive fluid management is advantageous over liberal fluid management in various surgical specialties [12,16]. Yet, very restrictive fluid management may also harm patients due to hypovolemia and its consequences [17]. As the evidence for fluid restriction in liver surgery is not yet well studied, we analyzed the outcomes of patients undergoing liver surgery at our center, where low-CVP and restrictive fluid management had been adopted.

Among 666 liver resections, we found no difference between the very restrictive and the normal fluid management groups in terms of postoperative morbidity, as determined by the CD-classification and the CCI. However, very restrictive fluid management was associated with a longer postoperative hospital and ICU length of stay and a higher postoperative mortality rate. In addition, increased lactate levels, higher doses of norepinephrine, the duration and extent of surgery, as well as red blood cell transfusion and estimated blood loss, were associated with the incidence of adverse events. In contrast, the last measured hemoglobin level and cumulative time of relative hypotension were not.

The effect of fluid therapy on postoperative morbidity increased in major/extreme procedures compared to the overall study population. Here, very low total and NFB levels and crystalloid fluid supplementation were associated with higher morbidity.

Subgroup analysis of patients with normal serum lactate levels showed that NFB and crystalloid administration were not associated with morbidity or ICU length of stay in this group.

The substantial sample size allowed for taking into account several potential confounders as covariates in the regression models, such as ASA status, age, sex, and, where appropriate, duration and size of intervention. These confounders are also known risk factors for postoperative complications that are also associated with postoperative intensive care [18]. Other patient- and intervention-specific risk factors were eliminated by applying exclusion criteria.

When statistically comparing baseline characteristics between the two fluid management groups, the large sample size must be taken into account. Here, statistical significance may result from differences that are small from a clinical perspective but can be demonstrated with high statistical power. In contrast to previous studies, intraoperative hypotension was not associated with morbidity in our series [19,20]. Hypotension was defined as a decrease in systolic blood pressure to <80% of baseline. As described by Sessler et al. [21] and Wesselink et al. [20], there is currently no consistent definition of hypotension in the literature. Our relative threshold for hypotension corresponds to the anaesthesiological intraoperative target in non-cardiovascular, non-cardiosurgical patients at our center and is one of the most commonly used thresholds in the literature. Due to the aforementioned variability in the definition of hypotension, we performed a further, more sensitive analysis using a cut-off of MAP ≤ 65 mmHg. Again, no significant association with postoperative CCI was found in these models. The CCI is more relevant than the individual complications scored by the CD score alone, as it includes all complications and reflects their severity.

Furthermore, a small, randomized trial of 48 patients undergoing major liver resection showed no difference in morbidity between goal-directed (restrictive) and the normal (liberal) fluid management regimen as assessed by CD-classification [22]. Unfortunately, the thresholds for both fluid management systems are not comparable to ours. In addition, this study may have been underpowered to detect such differences.

Postoperative surgical complications have a major impact on patients’ disability-free survival, increase costs, and are a major burden on the healthcare system [23,24,25]. The prevention of perioperative morbidity is therefore of paramount importance. However, objective assessment of surgical morbidity remains a challenge. The European Perioperative Clinical Outcome (EPCO) definitions [26] recommend the CD-classification [14] as the preferred grading system for individual adverse events due to its clear methodology and definitions: this classification scores the severity of complications according to their therapeutic consequences. Even with this classification, the complete assessment of surgical morbidity in the daily routine remains difficult, despite all efforts at standardization [27]. At least complications requiring intervention (°III-IV) can be reliably assessed, as most of these procedures are usually documented. The CD-classification is also the standard classification system used by the Department of General, Visceral, and Transplantation Surgery at the University Medical Centre of Mainz. In addition, we used the comprehensive complication index (CCI), which summarizes all complications of an individual patient according to the CD-classification and provides a continuous value between 0 and 100 as the worst (fatal) outcome. Hospital length of stay and mortality are objective parameters that can always be analyzed retrospectively. Due to their clinical importance, we chose morbidity, mortality, and hospital and ICU length of stay as endpoints for this analysis.

As expected, the extent and duration of surgery were independent risk factors for postoperative adverse events. Although we excluded procedures lasting less than 70 min, the effect of “frontloading” cannot be precluded: shorter procedures are associated with higher relative fluid doses as they are given during the induction period. This assumption is supported by higher crystalloid infusion rates in shorter procedures. In addition to intraoperative parameters, comorbidities and postoperative management may influence surgical morbidity. Due to the study design, we cannot completely exclude the effect of such parameters on our results.

With a CCI of 20.9 and a mortality rate of 4.5%, our results are well comparable with other large series on the outcome of liver surgery [28] and superior to the reported outcome in Germany [29], mainly due to the high degree of specialization and volume of the center.

Furthermore, the exclusion of high-risk patients by subgroup analysis suggests that optimization of perioperative management further improves outcomes. In our center, we have generally adopted a restrictive fluid policy, using mainly crystalloids for fluid replacement as recommended by the ERAS society [1]. In addition, patient blood management is being increasingly implemented, resulting in lower transfusion rates and increasing crystalloid infusions.

Moreover, these data should support a less dogmatic fluid management strategy in liver surgery. The aim of intraoperative fluid management is to support the surgical strategy of minimizing blood loss by reducing CVP, as this is a well-established outcome parameter in liver surgery [30]. Accordingly, avoiding fluid overload by restricting fluid infusion is beneficial, which is particularly evident in laparoscopic liver surgery, where a very restrictive fluid strategy is indeed anticipated by most surgeons in order to maintain a dry surgical field [31]. However, sudden events can dramatically destabilize a “dry” patient and place the patient at a particular risk during liver surgery.

The complex relationship between fluid administration and morbidity has been described as a U-shape, with the lowest morbidity in normovolemia and increased morbidity in hypo- and hypervolemia [13,17]. Our results support this U-shape hypothesis: after excluding patients with elevated lactate levels, fluid management was no longer associated with the endpoints of this analysis, and outcome was only related to the extent of surgery.

A wide range and variability in fluid management among patients undergoing liver resection have been shown to be multifaceted and influenced by factors at the patient, surgical, and provider levels [32]. Considering these general measures, only a very few patients met the criteria for a very liberal fluid replacement policy, and the vast majority would fit into the moderate range, while obviously some also met the very restrictive criteria proposed by Shin et al. [13]. Moreover, our fluid management is much lower than that reported by others [32].

Therefore, a more sophisticated strategy is required, and several parameters have been proposed to optimize intraoperative fluid management. However, the ideal parameters for optimal fluid management have not yet been identified. Moreover, calculated fluid balances between centers and publications are difficult to compare since different parameters have been included. In addition, some authors report total fluid administration or a total fluid balance, while others normalize for body weight and/or duration of anesthesia. Consequently, the crude parameters are difficult to compare. We provide a range of parameters that all demonstrate the same associations with our endpoints. Still, intraoperative fluid loss due to perspiration and blood, platelet, or fresh frozen plasma resuscitation has not been included in our fluid management due to the wide variation in volume included and the study design.

Serum lactate is a surrogate parameter for impaired tissue perfusion and a widely used outcome indicator in critically ill patients. Our data are consistent with the findings of Wiggans et al. [33].

The current trend in abdominal surgery is shifting towards goal-directed fluid management [34]. In liver surgery, the data are limited, and our work contributes to the re-evaluation of overly restrictive fluid management and a goal-directed strategy aiming at low euvolemia and restriction by normative lactate. The threshold of 10 mL kg^−1^ h^−1^ to define fluid management groups was based on heuristics and should be regarded as exploratory. Future studies will need to assess whether end-point-specific thresholds or possibly non-linear dose–response models for continuous fluid management better reflect the underlying mechanisms.

Due to the association between elevated lactate levels and outcomes, we are now using this parameter more intensively to control fluid management. Each center should decide on its own individual strategy to avoid peripheral hypoperfusion due to hypovolemia. Blood transfusions should also be used with caution in major liver resections, given the association with morbidity in our series.

Our study has several limitations. First, our study is retrospective, which limits our ability to control for confounding variables such as patient lifestyle factors and treatment compliance. These factors may have influenced the results we observed.

Second, our study was conducted at a single center, which may limit the generalizability of our findings.

Thirdly, we relied on electronic medical records to collect data on patient outcomes and intraoperative anesthetic management, so missing or incomplete information cannot be completely ruled out.

Finally, despite the large sample size of 666 patients, no power calculation was performed prior to the study, which may limit the statistical power of our findings.

## 5. Conclusions

In this retrospective study, the influence of intraoperative fluid management on postoperative morbidity in 666 patients undergoing liver surgery was assessed using the CD-score and CCI. Low NFB was associated with longer hospital and ICU lengths of stay and higher mortality. In the major extreme procedure subgroup, low NFB was associated with increased postoperative morbidity. In patients with normal lactate levels, there was no association between fluid management and morbidity.

Our results suggest that fluid management in liver surgery requires a more sophisticated approach to optimal fluid administration. Our study provides important insights into the relationship between fluid management and postoperative outcome in liver surgery and adds to the scarce literature in this area. Due to the apparent impact of intraoperative fluid management, further research in this area is required. Large-scale, standardized, preferably prospective, randomized controlled trials in patients undergoing liver surgery are needed to define the ideal parameter for fluid assessment and optimal intraoperative fluid management. Future standards should be based on the size of the procedure.

## Figures and Tables

**Table 1:** Patients’ characteristics.

Parameter	Overall	<10 mL·kg^−1^·h^−1^	≥10 mL·kg^−1^·h^−1^	*p*-Value
**Study population**	666	391 (58.71%)	275 (41.29%)	
**Age (years, mean ± SD)**	61.7 ± 12.9	61.8 ± 12.9	61.6 ± 13.1	0.71
**BMI (kg/cm², mean ± SD)**	26.8 ± 4.97	28.2 ± 4.93	24.9 ± 4.36	<0.001
**ASA I–II**	336 (50.5%)	176 (45.01%)	160 (58.18%)	<0.001
**ASA III–IV**	330 (49.5%)	215 (54.99%)	115 (41.82%)	0.031
**Diagnosis**				
**Hepatocellular carcinoma (HCC)**	123 (18.5%)	72 (18.4%)	51 (18.5%)	
**Hepatic adenocarcinoma**	26 (3.9%)	16 (4.1%)	10 (3.6%)	
**Extrahepatic Cholangiocarcinoma (CCC)**	62 (9.3%)	48 (12.3%)	14 (5.1%)	
**Intrahepatic Cholangiocarcinoma (CCC)**	67 (10%)	39 (10.0%)	28 (10.2%)	
**Colorectal metastases**	290 (43.5%)	165 (42.2%)	125 (45.5%)	
**Others**	98 (14.7%)	51 (13.0%)	47 (17.1%)	
**Extent of surgery**				
**Minor liver resections**	430 (64.6%)	238 (60.87%)	192 (69.82%)	0.031
**Major liver resections**	96 (14.4%)	58 (14.83%)	38 (13.82%)	
**Extreme liver resections**	140 (21.0%)	95 (24.3%)	45 (16.36%)	
**Duration of surgery (h, mean ± SD)**	4.29 ± 1.77	4.7 ± 1.81	3.71 ± 1.54	<0.001
**Anesthesia time (h, mean ± SD)**	5.78 ± 1.85	6.2 ± 1.91	5.2 ± 1.6	<0.001
**Estimated blood loss (ml, mean ± SD)**	1188 ± 1011	1127 ±910	1200 ± 1031	<0.001
**Red blood cell transfusion**	175 (26.3%)	27 (24.32%)	148 (26.67%)	0.012
**Total Norepinephrine (µg/kg ×** **min, mean ± SD)**	0.0416 ± 0.0408	0.040 ± 0.0381	0.043 ± 0.0444	0.64
**Maximum Lactate (mmol/L, mean ± SD)**	2.16 ± 1.37	2.33 ± 1.5	1.93 ± 1.13	<0.001
**Last Hemoglobin (g/dL, mean ± SD)**	10.7 ± 1.79	10.9 ± 1.84	10.5 ± 1.7	0.011
**Time of relative hypotension † (min, mean ± SD)**	138 ± 120	154 ± 126	116 ± 106	<0.001
**Sterofundin (ml·kg^−1^·h^−1^, mean ± SD)**	15 ± 6.13	11.9 ± 3.56	19.5 ± 6.22	<0.001
**Total Sterofundin volume (mL, min.–max.)**		4000 (1100–14500)	4500 (1500–24000)	0.015
**Clavien-Dindo = 0**	318 (47.75%)	170 (43.48%)	148 (53.82%)	
**Clavien-Dindo > 0**	348 (52.25%)	221 (56.52%)	127 (46.18%)	
**CCI-Score (mean ± SD)**	23.8 ± 26.0	26.4 ± 26.6	20.1 ± 24.9	
**Postoperative hospital LOS (days, mean ± SD)**	16.2 ± 15.0	17.5 ± 16.5	14.2 ± 12.3	
**ICU LOS (days, mean ± SD)**	2.96 ± 5.52	3.16 ± 5.99	2.64 ± 4.68	
**In-hospital mortality**	30 (4.5%)	20 (5.1%)	10 (3.64%)	

BMI: body mass index; LOS: length of stay; SD: standard deviation; † Relative hypotension: ≤80% baseline systolic arterial blood pressure. Minor liver resection: ≤3 segments; major liver resection: ≥4 segments; extreme liver resection: major resections with additional procedures).

**Table 2:** Odds Ratios for NFB in logistic regression models and hazard ratios for NFB in Cox regression models.

Endpoints	Odds Ratio	95% CI	*p*-Value
**Clavien-Dindo classification >/= 0**	1.0	0.97–1.04	0.89
**Mortality**	0.88	0.78–0.98	0.02
	**Hazard Ratio**	**95% CI**	***p*** **-value**
**Postoperative hospital LOS**	1.05	1.02–1.09	<0.001
**ICU LOS**	1.04	1.00–1.08	0.035

CI: Confidence Interval; ICU: Intensive Care Unit; LOS: length of stay (Models for Clavien-Dindo classification >/= 0, postoperative LOS and ICU LOS adjusted for sex, age, extent as well as duration of surgery, crystalloid infusion, last hemoglobin-level (<10 vs. ≥10 g/dL), highest intraoperative lactate level (<1.5 vs. ≥1.5 mmol∙L^−1^), norepinephrine, red blood cell transfusion, estimated blood loss, time of relative hypotension (<100 vs. ≥100 min), and ASA physical status (I/II vs. III/IV) Mortality model adjusted for age, extent as well as duration of surgery, and crystalloid infusion).

**Table 3:** Predictive factors (Hazard Ratios) in Cox regression models for postoperative hospital and ICU stays.

Intraoperative Parameters	Hazard Ratio	95% CI	*p*-Value
**Postoperative hospital LOS**			
**Extent of surgery**	0.66	0.52–0.84	<0.001
**Duration of surgery**	0.79	0.75–0.84	<0.001
**Total Fluid balance (TFB)**	1.15	1.05–1.26	0.004
**Normalized fluid balance (ml kg^−1^ h^−1^) (NFB)**	1.053	1.02–1.09	<0.001
**Crystalloid infusion**	0.88	0.81–0.96	0.004
**ICU LOS**			
**Intraoperative lactate**	0.72	0.56–0.92	0.008
**Duration of surgery**	0.85	0.8–0.92	<0.002
**Normalized fluid balance (ml kg^−1^ h^−1^) (NFB)**	1.04	1.003–1.08	0.035

CI: Confidence Interval; ICU: Intensive Care Unit; LOS: length of stay (All models adjusted for Clavien-Dindo classification >/= 0, postoperative LOS and ICU LOS adjusted for adjusting for sex, age, extent as well as duration of surgery, crystalloid infusion, last hemoglobin-level (<10 vs. ≥10 g/dL), highest intraoperative lactate level (<1.5 vs. ≥1.5 mmol∙L^−1^), norepinephrine, red blood cell transfusion, estimated blood loss, time of relative hypotension (<100 vs. ≥100 min), and ASA physical status (I/II vs. III/IV)).

**Table 4:** Association of clinical parameters with the comprehensive complication index (CCI) and mortality from linear regression models.

Exploratory Risk Factor	Estimate	t Value	*p*-Value
**Morbidity**			
**Age**	0.34	4.77	<0.001
**Sex**	−1.81	−0.95	0.343
**Red blood cell transfusion**	2.70	6.02	<0.001
**Estimated blood loss**	5.41	5.14	<0.001
**Relative Hypotension**	0.009	1.06	0.29
**Lactate**	2.59	3.54	<0.001
**Norepinephrine (µg kg^−1^ min^−1^)**	53.14	2.34	0.02
**Duration of surgery**	5.19	8.47	<0.001
**Hb last**	−1.24	−2.30	0.022
	**Odds Ratio**	**95% CI**	***p*** **-value**
**Mortality**			
**Age**	1.03	0.99–1.06	0.119
**Extent of surgery**	4.94	2.21–12.12	<0.001
**Total fluid balance (TFB)**	1.23	1.03–1.24	0.01
**Normalized fluid balance (NFB)**	0.88	0.78–0.98	0.023

Hb, Hemoglobin; CI, Confidence Interval.

## Data Availability

The data, as well as the study protocol, are available from the authors upon request.
